# 2448. Emergence of *Candida auris* in a Delaware Healthcare System

**DOI:** 10.1093/ofid/ofad500.2066

**Published:** 2023-11-27

**Authors:** Rebecca Pheiffer, Carol Briody, Ken Anderson, Elicious Cyril, Lajune Harris, Timothy Heckman, Stephanie Kelly, Kathleen Luckner, Julia Moeller, Carmen Pal, Ryan Penn, Michelle Power, Carol Ritter, Ann-Catherine Stanton, Marci Drees

**Affiliations:** ChristianaCare, Wilmington, Delaware; ChristianaCare, Wilmington, Delaware; ChristianaCare, Wilmington, Delaware; ChristianaCare, Wilmington, Delaware; Delaware Division of Public Health, Dover, Delaware; ChristianaCare, Wilmington, Delaware; ChristianaCare, Wilmington, Delaware; ChristianaCare, Wilmington, Delaware; ChristianaCare, Wilmington, Delaware; ChristianaCare, Wilmington, Delaware; ChristianaCare, Wilmington, Delaware; ChristianaCare, Wilmington, Delaware; ChristianaCare, Wilmington, Delaware; Delaware Division of Public Health, Dover, Delaware; ChristianaCare, Wilmington, Delaware

## Abstract

**Background:**

In Aug 2022, a patient was admitted to our facility after blood cultures revealed *C. auris*; this was the first case identified in Delaware. The patient had been cared for in our system 2 mo previously and was admitted from a long-term acute care hospital. In Sep 2022, two additional cases were identified who had been recently or were currently admitted to our transitional medical (stepdown) unit (TMU).

**Methods:**

We worked with the Delaware Division of Public Health (DPH) to establish protocols to identify colonized cases via biweekly point prevalence (PP) surveys of all patients currently admitted on TMU using the public health laboratory; later we transitioned to weekly PP via a commercial lab to improve frequency and turnaround time. Patients were screened using axillary/groin swabs. We provided ongoing staff education; improved adherence to hand hygiene and contact isolation precautions; created auto-isolation orders for newly identified and readmitted patients; and reviewed disinfectants. Environmental Services (EVS) performed enhanced cleaning of all patient rooms and auxiliary areas on the unit, and added ultraviolet light and curtain changes for all terminal cleans. DPH performed an Infection Control Risk Assessment (ICAR) to identify further opportunities. We expanded these interventions to our medical ICU (MICU) when cases were identified there, and added admission surveillance screening of high-risk patients to TMU or MICU.

**Results:**

Between Aug 2022 – Mar 2023, 64 patients with *C. auris* were identified (55 [86%] via PP surveys) (Table). Greater than 94% of eligible patients were successfully screened each week; PP positivity rate ranged from 0-30% (Fig). Admission screening continued to identify new cases admitted to these units. Four (6.5%) patients initially identified with *C. auris* colonization developed clinical infections.

**Figure:**
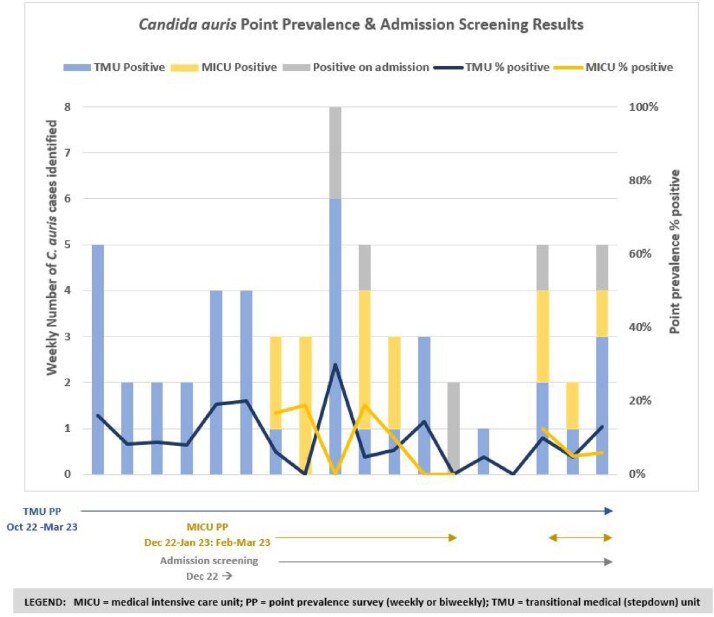

Characteristics of Patients Identified with Candida auris Colonization or Infection

**Figure:**
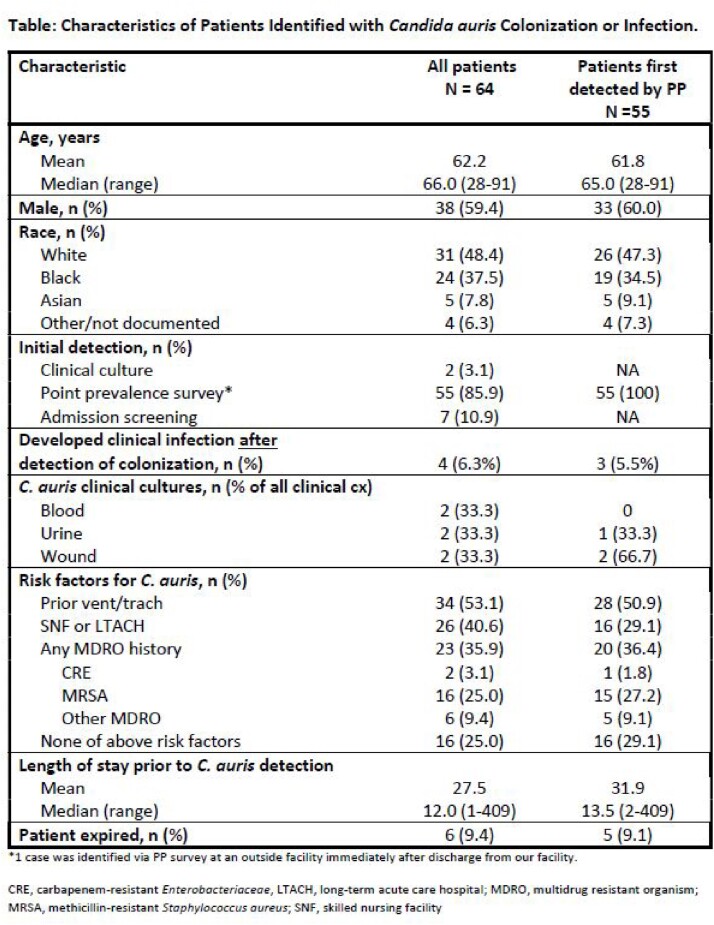

**Conclusion:**

Despite expending significant lab, EVS and other personnel resources to improve patient identification and infection control practices, once *C. auris* was established in the units it was unable to be eradicated. These resources were not sustainable; we are shifting from PP to expanded admission screening while maintaining environmental cleaning and infection prevention practices.

**Disclosures:**

**All Authors**: No reported disclosures

